# Arsenic efflux mechanisms in ectomycorrhizal mushrooms *Hebeloma bulbiferum* and* Hebeloma sinapizans*

**DOI:** 10.1007/s00253-026-13710-7

**Published:** 2026-01-30

**Authors:** Jan Šnábl, Gabriela Pelešková, Antonín Kaňa, Martina Šnáblová, Tereza Leonhardt, Jan Borovička, Jan Sácký

**Affiliations:** 1 Department of Biochemistry and Microbiology, University of Chemistry and Technology, Prague, Technická 3, 166 28, Prague, Czech Republic; 2 Czech Academy of Sciences, Nuclear Physics Institute, Hlavní 130, 25068 Husinec-Řež, Czech Republic; 3 Czech Academy of Sciences, Institute of Geology, Rozvojová 269, 16500 Prague 6, Prague, Czech Republic; 4 Department of Analytical Chemistry, University of Chemistry and Technology, Prague, Technická 5, 166 28 Prague, Czech Republic

**Keywords:** Arsenic transport, Fungi, ACR3 transporters, *Agaricales*, Mushrooms

## Abstract

**Abstract:**

Arsenic (As) is a toxic metalloid widespread in the environment, and many organisms have evolved mechanisms to mitigate its toxic effects. Bioinformatic analyses revealed that *acr3* genes are predominantly distributed in mushrooms, highlighting their evolutionary and functional importance in eukaryotic arsenic metabolism. In this study, two homologous genes, Hb*ACR3* and Hs*ACR3*, from the mushrooms *Hebeloma bulbiferum* and *Hebeloma sinapizans* were identified and functionally characterized. Both encode 399-amino-acid membrane proteins showing 99% sequence identity to each other and substantial similarity to previously characterized ACR3-type arsenite transporters from plants, yeasts, and bacteria. Heterologous expression of Hb*ACR3* and Hs*ACR3* in a *Saccharomyces cerevisiae arr3*Δ mutant restored resistance to arsenite and arsenate and significantly reduced intracellular arsenic accumulation. Fluorescence microscopy of GFP-tagged Hb*ACR3* and Hs*ACR3* confirmed their localization to the plasma membrane, consistent with an efflux transport function. Exposure of *H. bulbiferum* and *H. sinapizans* mycelia to arsenate led to a significant but differential transcriptional upregulation of both genes*.* This work provides new insight into the evolution, distribution, and physiological significance of ACR3 transporters in eukaryotic arsenic homeostasis.

**Key points:**

- *Acr3 genes are widespread in fungi, indicating a key role in arsenic detoxification.*
- *HbACR3 and HsACR3 reduce cellular arsenic and confer As(III) tolerance.*
- *Arsenate exposure strongly induces HbACR3 and HsACR3 gene expression.*

**Supplementary Information:**

The online version contains supplementary material available at 10.1007/s00253-026-13710-7.

## Introduction

Arsenic is a trace element (metalloid) most renowned for its toxic effects (Wenzel 2013). When disturbed by natural processes (weathering) or anthropogenic activities (mining and smelting operations, coal combustion), arsenic can be released into the environment, where it may pose a threat to living organisms (Shen et al. 2013). Arsenic naturally occurs in a variety of chemical forms. Among them, the inorganic forms are the predominant species found in the terrestrial environments, and in general, inorganic arsenic (iAs) is significantly more toxic to humans than organic arsenicals (Sun et al. 2015). Arsenate As(V) can compete with the essential inorganic phosphate, and arsenite As(III) can inactivate numerous enzymes by binding to protein thiols. Therefore, arsenic detoxification pathways are presented in nearly all organisms, from bacteria to humans. Generally, within the cell, arsenate As(V) is initially reduced to arsenite As(III) by arsenate reductase (Dhankher et al. 2006; Mukhopadhyay and Rosen 1998; Jia et al. 2019; Popov et al. 2021), and the resulting As(III) is subsequently removed from the cytoplasm through specific efflux transporters. This process is regarded as a key mechanism of arsenic detoxification in both prokaryotic and eukaryotic organisms (Maciaszczyk-Dziubinska et al. 2012; Yang et al. 2015; Garbinski et al. 2019). Another important mechanism involves the biotransformation of inorganic arsenic into organic arsenicals. This process includes redox enzymes, methyltransferases, and biosynthetic pathways responsible for the production of arsenosugars, arsenolipids, and other less or non-toxic arsenic forms.

Six types of specific arsenic efflux transporters (ArsB, ACR3, ArsJ, ArsP, ArsK, and MSF1) have been characterized to date (Achour et al. 2007; Garbinski et al. 2019). The major protein families, ArsB and ACR3, form a group of arsenite efflux pumps that mediate the export of iAs, but not organoarsenic species. While ArsB and ACR3 transporters share the same function, they evolved independently and exhibit minimal sequence similarity (Mansour et al. 2007; Garbinski et al. 2019). ArsB is only found in prokaryotes, whereas ACR3 proteins are widely distributed not only in bacteria and archaea, but also in yeast and some plants (Mansour et al. 2007; Castillo and Saier 2010; Yang et al. 2015; Garbinski et al. 2019). The ArsK family also confers resistance to As(III), as well as to methylarsenite MA(III). Genome analyses have shown that ArsK orthologs are widely distributed in arsenic-resistant bacteria but are absent in archaea, fungi, plants, and animals (Shi et al. 2018; Garbinski et al. 2019). Another bacterial transporter family, ArsJ, confers resistance to As(V) but not to As(III), and it is the only known efflux permease that exports pentavalent arsenic (Chen et al. 2016). Originally reported as an organic arsenic transporter for pentavalent forms, ArsP was later shown to have a specificity for trivalent organic arsenicals (Shen et al. 2014; Chen et al. 2015).

The ACR3 protein family (TCDB: 2.A.59) belongs to the bile/arsenite/riboflavin transporter (BART) superfamily and functions as a metalloid/antiporter. The *S. cerevisiae* homolog ScACR3 (ScARR3) is currently the only characterized fungal member and, unlike bacterial orthologues described to date which are restricted to As(III), is capable of transporting both As(III) and Sb(III) (Wysocki et al. 1997). Substrate recognition in ScACR3 relies on motifs distributed across 10 transmembrane domains (TMDs), most notably a critical cysteine residue in TMD4. Additional residues within TMD5 and TMD9 contribute to a structural crossover region (involving TMDs 4/9 and 5/10) proposed to mediate substrate binding and As(III)/H^+^ exchange (Mizio et al. 2023).

Despite the significant role fungi play in biogeochemical cycling, their specific contribution to terrestrial arsenic turnover remains largely underexplored. Although ACR3-mediated extrusion of As(III) is the most prevalent tolerance mechanism in both prokaryotes and eukaryotes (Maciaszczyk-Dziubińska et al., 2012 ; Markowska et al. 2015; Yang et al. 2015) and is likely a source of naturally occurring As(III), no ACR3 transporters from mushrooms, also known as macrofungi, have been reported or characterized to date. The present study aimed to identify *acr3* genes in fungi, particularly in two ecologically and phylogenetically close species displaying distinct arsenic speciation profiles, *Hebeloma bulbiferum* and *Hebeloma sinapizans*. We characterized the genes encoding functional HbACR3 and HsACR3 transporters. When expressed in model yeast, both proteins enhanced tolerance to As(III) and As(V), while significantly decreasing total arsenic accumulation. Additionally, in mycelial isolates of *H. bulbiferum* and *H. sinapizans*, exposure to arsenate As(V) strongly induced the expression of both Hs*ACR3* and Hb*ACR3*, highlighting their key role in arsenic detoxification in these fungi.

## Materials and methods

### Mushrooms, yeasts, and general procedures

Fruit bodies of *H. bulbiferum* and *H. sinapizans* and surrounding soil were collected at a *Carpinus betulus* plantation on Silurian limestone at Prague–Velká Chuchle, Czechia. The dikaryotic mycelial isolates of *H. bulbiferum* and *H. sinapizans* were prepared from the axenic explants from the freshly broken fruit body flesh of both investigated species, as described previously (Osobová et al. 2011). The identity of the isolated *H. bulbiferum* and *H. sinapizans* strains, BULB1 and SINA1, respectively, was confirmed by ITS rDNA sequencing, and both sequences were deposited in the National Center for Biotechnology, GenBank (accessions PV902627.1 and PV747898.1, respectively). The mycelial isolates were stored and maintained on half-strength potato dextrose (PD) agar plates (6 g L^−1^ PD broth; 10 g L^−1^ agar, Formedium).

The plasmid p416GPD used in this study is the same as that described by Sácký et al. (2025), where the composition of the synthetic defined (SD) medium lacking uracil is also provided. For heterologous expression of *ACR3*s and their GFP variants, we used the metal-sensitive *Saccharomyces cerevisiae* URA3⁻ mutant *arr3*Δ (BY4741 background; *MATa his3*Δ*1 leu2*Δ*0 met15*Δ*0 ura3*Δ*0* YPR201w::kanMX4) obtained from Euroscarf. The isogenic BY4741 strain served as an arsenic-tolerant control.

### Identification, PCR amplification, and cloning of ACR3 cDNAs

The identification of *ACR3* genes in *H. bulbiferum* and *H. sinapizans* was based on BLASTp searches of the genome sequence-derived proteome of the closely related *Hebeloma cylindrosporum* at the UNIPROT site, with the target database set to UniProt reference proteomes + Swiss-Prot, restricted to *H. cylindrosporum h7* (Doré et al. 2015, 2017; Kohler et al. 2015). The query used was the previously characterized *S. cerevisiae* ACR3 (GenBank: AAB64629.1). The returned putative protein sequence A0A0C2XVF8 was used to retrieve the corresponding genomic sequences from the *H. cylindrosporum h7* v2.0 genome hosted at JGI, MycoCosm (Grigoriev et al. 2014).

The *ACR3* genes and their coding sequences from *H. bulbiferum* and *H. sinapizans* mycelia were amplified from genomic DNA and cDNA obtained as described by Sácký et al. (2025). Briefly, genomic DNA was isolated from 25 mg of freeze-dried sporocarp tissue by using the NucleoSpin Plant II Kit (Macherey-Nagel) according to the manufacturer’s instructions; total RNA was isolated from 25 mg of freeze-dried sporocarp tissue by using the RNeasy Plant Mini Kit (Qiagen) with buffer RLT according to the manufacturer’s instructions and converted to cDNA using the High-Capacity cDNA Reverse Transcription Kit (Applied Biosystems) with 0.5 μg of the total RNA according to the manufacturer’s instructions. Complete genomic sequences and their flanking regions were obtained by homology cloning using the GenomeWalker Universal Kit (Clontech) with primers listed in Supplementary Table 1. Target genomic DNA and cDNA sequences were amplified by PCR using primers from Supplementary Table 1, and *ACR3* cDNAs were cloned into BamHI-linearized p416GPD (Mumberg et al. 1995) and p416GFP (Hložnová et al. 2016) vectors via NEBuilder® HiFi DNA Assembly (New England Biolabs), transformed into *E. coli* DH5α, grown in Luria-broth medium with 100 µg mL^−1^ ampicillin. Isolated plasmids were sequenced (by Eurofins) to confirm the cDNA sequences and further used in the *S. cerevisiae* experiments. GenBank accession numbers PV925732.1 and PV925734.1 correspond to the genomic sequences of Hb*ACR3* and Hs*ACR3*, respectively. The amino acid sequences inferred from the cDNAs were cross-checked via reciprocal BLASTp using UniProt databases.

### Database search for ACR3 homologs

Homologs of ACR3 were searched in the NCBI database using BLASTp with default settings, and individual groups of organisms were always selected as the target database. Furthermore, the phylogenetic distribution of ACR3 family proteins was searched within the InterPro database (v 103.0).

### Functional complementation and accumulation assays in *S. cerevisiae*

For the As tolerance plate assays, the mid-log cultures of *S. cerevisiae* transformants were adjusted to an optical density at 590 nm (OD_590_) of 0.2, followed by preparation of tenfold serial dilutions. A 4 µL aliquot of each dilution was then spotted onto SD agar plates either without metal addition or supplemented with NaAsO₂ to a final concentration of 250 µM and 1000 µM, or with Na₂HAsO₄·7 H₂O to a final concentration of 250 µM and 500 µM.

Arsenic uptake assays in SD medium were initiated by adding the appropriate concentrations of NaAsO₂ to 40 mL of the yeast cultures that reached OD_590_ of 1.6–1.8, and the cultures were further agitated for 1 h at 30 °C at 150 rpm. The cells were then harvested by centrifugation at 3 000 × g and 25 °C for 2 min and washed twice with 20 mL of 50 mM phosphate buffer (pH 6.5) for 5 min.

The sample for total As determination was subjected to microwave-assisted digestion in a Teflon® vessel with 3 mL of concentrated nitric acid (Analpure grade, Analytika, Czech Republic). The temperature was ramped to 240 °C within 8 min and held at that temperature for 5 min. The digest was then quantitatively transferred into a 50-mL volumetric flask. The total arsenic and its species content in the mushrooms, mycelia, and in the yeasts was determined with inductively coupled plasma mass spectrometry (ICP-MS), following a previously developed method (Kaňa et al. 2020). The mobile phase (flow rate 1.0 mL·min⁻^1^) was delivered by a PerkinElmer Series 200 HPLC pump and consisted of 1.0 g sodium butane-1-sulfonate (> 99.0%, Sigma-Aldrich, St. Louis, USA), 0.42 g malonic acid (for synthesis, Merck, Darmstadt, Germany), 0.22 g tetramethylammonium hydroxide pentahydrate (≥ 97%, Sigma-Aldrich, St. Louis, USA), and 500 μL methanol (Penta s.r.o., Prague, Czech Republic) dissolved in 1 L of demineralized water. Element-specific detection was performed using an ICP-MS NexION 350D (PerkinElmer, Concord, Canada), operated with ammonia (grade 5.0, SIAD, Czech Republic) as a reaction gas (flow rate 0.3 mL·min⁻^1^) to eliminate ArCl⁺ interferences.

### Fluorescence microscopy

Fluorescence microscopy was performed as described by Sácký et al. (2025). Briefly, yeast cells transformed with the p416GFP-derived plasmids containing Hb*ACR3* or Hs*ACR3* were grown to an OD_590_ of 2, separated by centrifugation, and labeled using the fluorescent dye FM4-64 (Vida a Emr, 1995; Hickey et al. 2004) for 12 h at room temperature, mounted on glass slides in fresh SD medium, and immediately observed. GFP- and FM4-64-derived fluorescence was observed with FITC/Cy2 and TRITC/Cy3 filters, respectively. Images were obtained with an Olympus BX53 microscope equipped with a DP74 digital camera.

### Relative quantification of Hb*ACR3 *and Hs*ACR3*

The As(V) responsiveness of Hs*ACR3* and Hb*ACR3* was assessed by quantitative real-time PCR (RT-qPCR) using the same procedures as described by Sácký et al. (2025) with *ACR3*-specific primers (Supplementary Data 1). Mycelia were pre-grown for 21 days in PD medium before 24-h exposure to arsenate (Na₂HAsO₄·7 H₂O). Total RNA was isolated from 20 mg of lyophilized, ground mycelia using the RNeasy Plant Mini Kit (Qiagen), including on-column DNase digestion (Qiagen). Subsequently, cDNA was synthesized from 0.25 μg of total RNA using the High-Capacity cDNA Reverse Transcription Kit (Applied Biosystems). The β-tubulin genes, Hb*TUB2* and Hs*TUB2* (GenBank accession numbers PV925733 and PV925735, respectively), were used as reference genes. Amplification efficiencies were calculated as 93 % for Hs*ACR3*, 94 % for Hb*ACR3*, 107 % for Hs*TUB2*, and 106 % for Hb*TUB2*. Relative ACR3 transcript abundance was quantified using the 2^−ΔΔct1^ method (Pfaffl 2001). All experiments were conducted using three independent biological replicates, each measured in technical duplicate.

### Statistical analyses

Data for both the metal accumulation (Fig. 4) and gene expression (Fig. 6) assays are presented as means ± standard deviation (SD). Statistical significance was determined using Statistica 14.0 via a one-way ANOVA (*p* < 0.05) followed by Tukey’s post-hoc test to separate homogeneous groups.

## Results

This study focused on two closely related fungal species, *H. bulbiferum* and *H. sinapizans*, which exhibit distinct capacities for arsenic accumulation. Although both species were collected from the same site, *H. bulbiferum* (355 mg As kg^−1^ dry weight) showed an approximately 20-fold higher As accumulation than *H. sinapizans* (16 mg As kg^−1^ dry weight). Given the lack of information on arsenic tolerance and detoxification mechanisms in fungi, this process warranted a detailed investigation, which we specifically focused on ACR3 transporters as key mediators of arsenic resistance mediated by efflux.

### In silico characterization of Hb*ACR3* and Hs*ACR3*

**Fig. 1:**
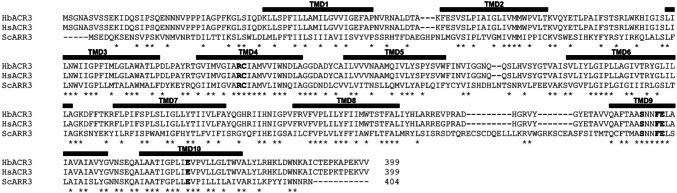
Sequence comparison of HbACR3 from *Hebeloma bulbiferum*, HsACR3 from *Hebeloma sinapizans*, and ScARR3 from *Saccharomyces cerevisiae*. The alignment of HbACR3 (XXQ47976), HsACR3 (XXQ47978), and ScARR3 (AAB64629) protein sequences (GenBank identifiers). The positions of the predicted transmembrane domains (TMDs) are noted by black boxes. The residues previously identified in ACR3 as important for transport activity are indicated within TMDs in bold

The predicted Hb*ACR3* and Hs*ACR3* cDNAs encode a protein of 399 amino acids, which are 99% identical. Both HbACR3 and HsACR3 proteins exhibit features characteristic of ACR3 proteins, including ten predicted transmembrane domain (TMD) helices, a highly conserved cysteinyl residue within the P/R-C-T/I-AMV motif in TMD3/4, and a conserved glutamatyl residue in TMD9/10 (Fig. 1).

**Fig. 2:**
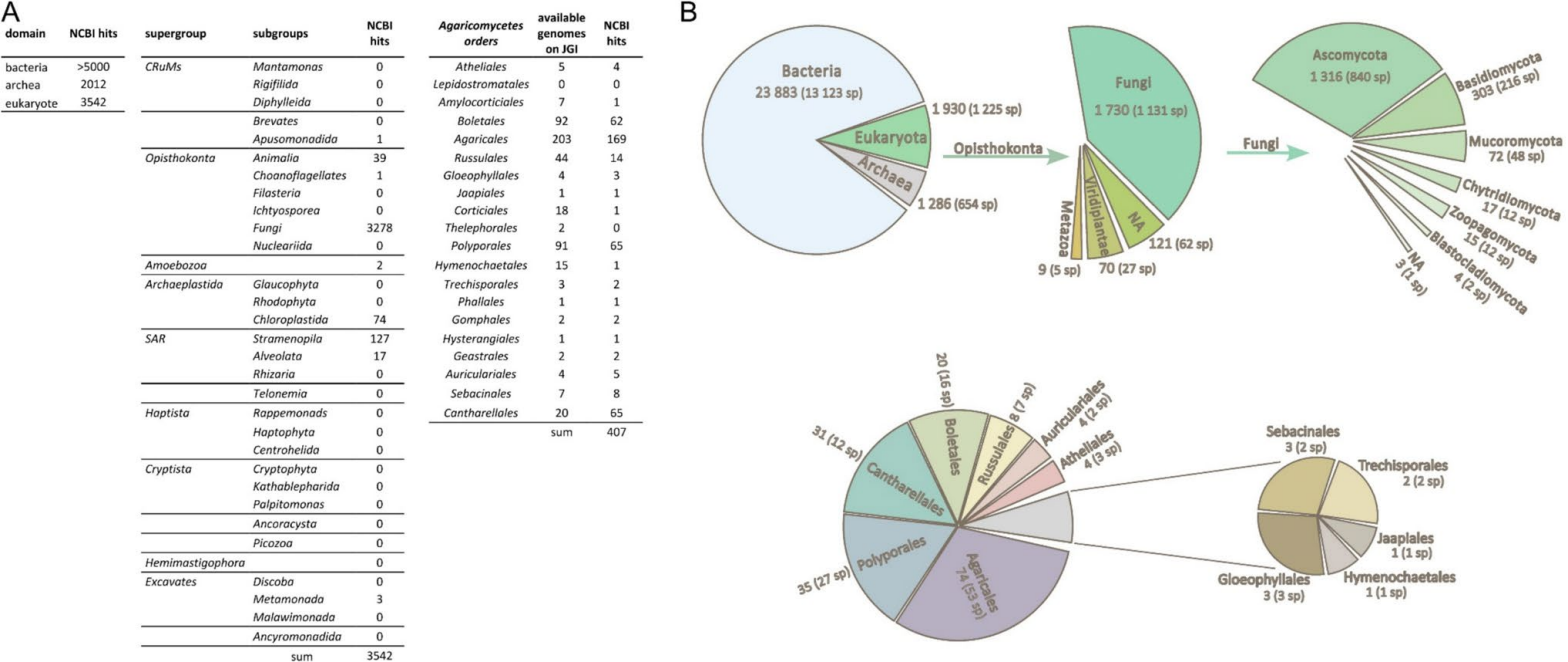
The taxonomic distribution of ACR3 homologs across domains of life. This analysis was performed following the latest molecular phylogenetic framework (Burki et al. 2020). **A** Table summarizing the number of ACR3 protein homologs identified via NCBI BLAST across major domains, including detailed counts across eukaryotic supergroups and fungal orders within the *Agaricomycetes* class. **B** Phylogenetic breakdown of proteins from the ACR3 family according to the InterPro database (v 103.0) homolog hits. The expanded view highlights distribution within the *Fungi* domain and the class *Agaricomycetes*, showing the number of hits and the number of species carrying the sequences

BLASTp analysis of NCBI databases revealed that HbACR3 and HsACR3 orthologs are widely distributed (Fig. 2A), with over 5000 hits in *Bacteria*, 2012 in *Archaea*, and 3542 in *Eukaryota*. Among the eukaryotic hits, 3278 (92.5 %) were found in fungi, while only a small number of orthologs were identified in other phylogenetic groups, including *Apusomonadida* (1 hit), *Animalia* (39 hits), *Choanoflagellates* (1 hit), *Amoebozoa* (2 hits), *Chloroplastida* (74 hits), *Stramenopila* (127 hits), *Alveolata* (17 hits), and *Metamonada* (3 hits) (Fig. 2A). Comparative BLASTp analysis confirmed that HbACR3 and HsACR3 orthologs are ubiquitous among *Agaricomycetes* (Fig. 2A). Also, in the InterPro database (v 103.0), most proteins from the arsenical-resistance protein family ACR3 are distributed in domain bacteria (88 %) (Fig. 2B). Within the *Opisthokonta* eukaryotic clade, the kingdom *Fungi* is the only dominant group carrying the ACR3 protein (with 90 % of the sequences), the presence of which is rare in other eukaryotic kingdoms. Among *Fungi*, most ACR3 sequences are carried by the members of phyla *Ascomycota* (76 %), followed by *Basidiomycota* (18 %). Taken together, these findings suggest that *ACR3* genes in eukaryotes are present predominantly in fungi.

### Functional characterization of *ACR3s *in* S. cerevisiae arr3*Δ

**Fig. 3:**
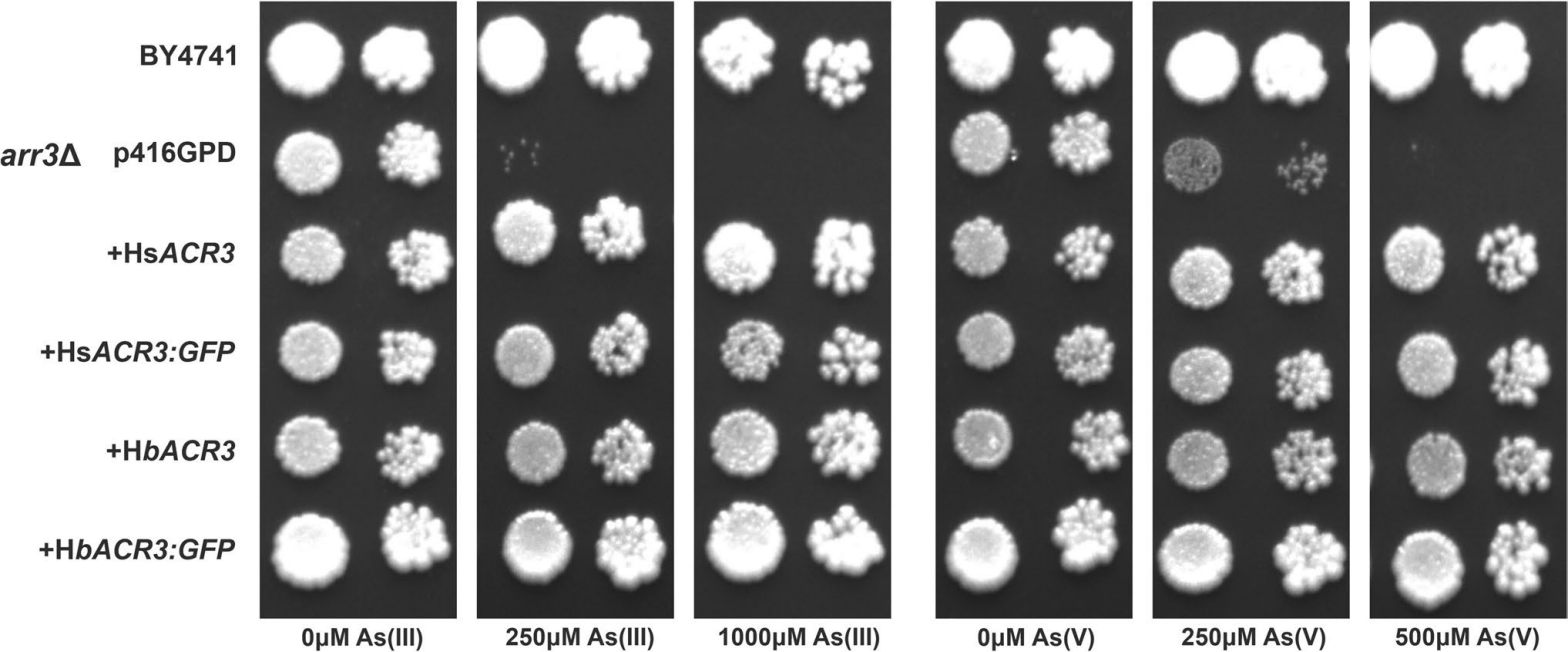
Arsenic tolerance of *Saccharomyces cerevisiae arr3*Δ metal-sensitive strains expressing Hs*ACR3* and Hb*ACR3* and their GFP variants. The p416GPD transformed BY4741 strain was used as a wild-type control. Cultures of wild type and mutant yeast were tenfold serially diluted and spotted on control and arsenic-supplemented SD medium

Alignment analysis predicted that Hb*ACR3* and Hs*ACR3* genes encode membrane As(III) transporters. To further investigate their transport activity, the corresponding coding sequences were inserted into the centromeric yeast vector p416GPD, and they were expressed in *S. cerevisiae arr3*Δ mutant. The wild-type BY4741 strain, transformed with the empty p416GPD vector, was used as a control. Transformed yeasts were thus grown on solid SD medium with or without As(III) and As(V) to assess the dose-dependent inhibitory effects of these arsenic compounds. When expressed in the *arr3*Δ cells, both Hb*ACR3* and Hs*ACR3* genes fully complemented the As-sensitive phenotype on agar plates supplemented with 250 μM As(III) and 500 μM As(V) (Fig. 3).

### Arsenic uptake of the yeasts expressing *ACR3* cDNAs

**Fig. 4:**
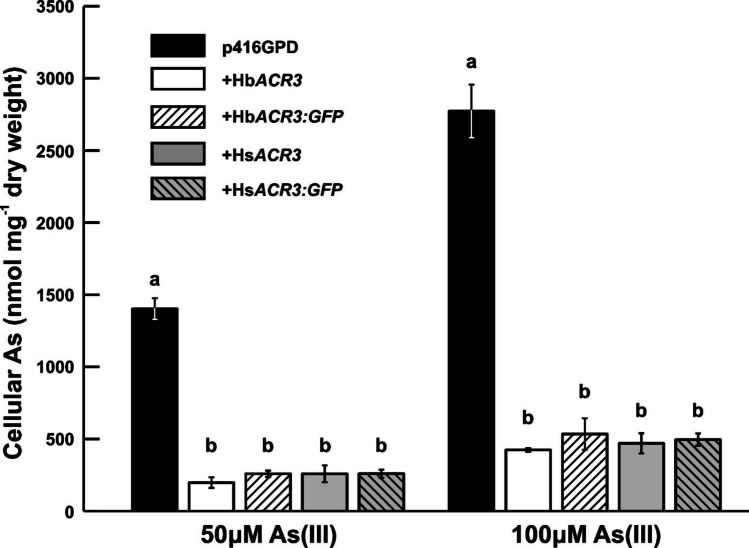
The accumulation of total arsenic in Hs*ACR3*-, Hb*ACR3-*, and their GFP variants-expressing *Saccharomyces cerevisiae arr3*Δ. The arsenic content of the *ACR3*-expressing and control p416GPD-transformed cells propagated in SD medium was measured following a 1-h incubation in the presence of the indicated concentrations of arsenic species. The plotted values represent the average of three biological replicates, error bars represent standard deviation, and significant differences (*p* < 0.05, ANOVA followed by Tukey’s HSD test) in each treatment are indicated by different letters above the bars

Since the expression of Hs*ACR3* and Hb*ACR3* conferred increased arsenic tolerance in the *S. cerevisiae arr3*Δ strain, we hypothesized that they act either as plasma membrane efflux transporters or as vacuolar transporters facilitating arsenic compartmentalization. To distinguish between these possibilities, we measured intracellular arsenic accumulation in *S. cerevisiae arr3Δ* cells incubated for 1 h in SD medium supplemented with As(III). Yeast cells expressing Hs*ACR3* and Hb*ACR3* accumulated statistically significantly lower amounts of As(III) compared to the p416GPD-transformed control cells. At 50 µM As(III), accumulation was reduced on average to 18 % for Hs*ACR3* and 14 % for Hb*ACR3*, while at 100 µM As(III), it was reduced to 17 % for Hs*ACR3* and 15 % for Hb*ACR3* (Fig. 4).

### Localization of Hb*ACR3* and Hs*ACR3* in yeast model

**Fig. 5:**
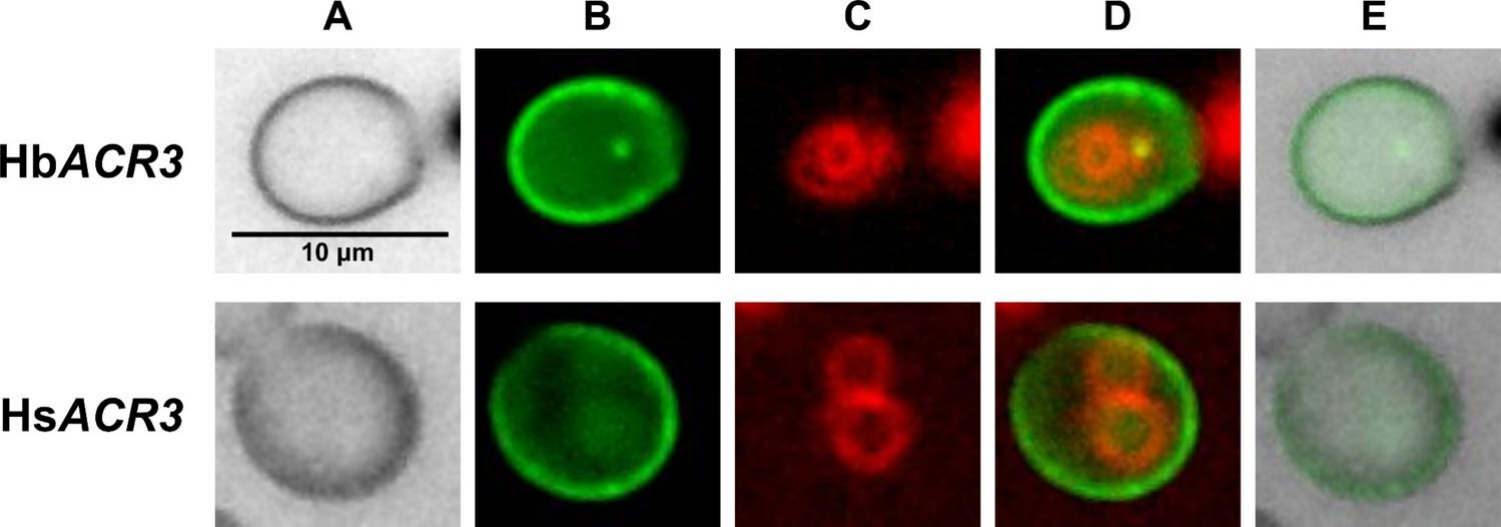
Localization of the HbACR3:GFP and HsACR3:GFP fusion proteins in the *Saccharomyces cerevisiae arr3*Δ strain. **A** Bright field image. **B** GFP fusion protein (green fluorescence). **C** FM4–64 vacuolar membrane staining (red fluorescence). **D** Merged images (**B**) and (**C**). **E** Overlay image of (**A**) and (**B**), indicating green fluorescence on cytoplasmic membrane, in the close vicinity of cell wall

As the expression of Hb*ACR3* and Hs*ACR3* reduced arsenic accumulation in yeast, we tested whether they function as plasma membrane transporters by tagging them with GFP and analyzing their localization. Their distribution was examined in yeast cells using the lipophilic dye FM4-64 as a vacuolar membrane marker. Fluorescence microscopy of the yeasts expressing Hb*ACR3:GFP* and Hs*ACR3:GFP* revealed clear GFP signals at the cell periphery (Fig. 5), indicating that HbACR3 and HsACR3 are localized to the plasma membrane. Furthermore, both Hb*ACR3:GFP* and Hs*ACR3:GFP* were functionally active, as they fully complemented the metal sensitivity of *arr3*Δ cells and exhibited a reduced arsenic accumulation phenotype just like their non-tagged counterparts (Figs. 3 and). These results confirm that the GFP fusion proteins retained their original function and that the observed localization is biologically relevant.

### Hb*ACR3* and Hs*ACR3* expression in mycelia upon arsenic exposure

**Fig. 6:**
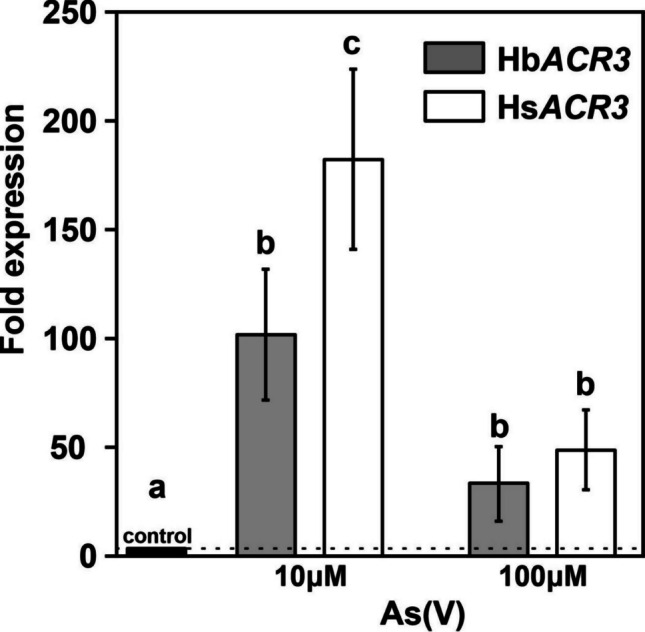
The relative expression of Hb*ACR3* and Hs*ACR3* in *Hebeloma bulbiferum* and *Hebeloma sinapizans* mycelia exposed to excess As(V). The treatments were started by adding As(V) to the medium, and mycelia were incubated for 24 h. Gene expression was measured by RT-qPCR and is presented as fold changes (arsenic-exposed relative to non-exposed control). Genes coding for beta tubulin (Hb*TUB2* and Hs*TUB2*) were used as controls. The plotted values represent the average of three biological replicates, error bars represent standard deviation, and significant differences (*p* < 0.05, ANOVA followed by Tukey’s HSD test) in a particular treatment are indicated by different letters above the bars

Given that Hb*ACR3* and Hs*ACR3* function as arsenite efflux permeases, their expression is likely important for handling intracellular arsenic and dependent on arsenic exposure. Since As(V) reduction activity has been observed in both mycelia of *H. bulbiferum* and *H. sinapizans* (Supplementary Data S1), we analyzed their transcriptional response to arsenate. Exposure to As(V) markedly upregulated the transcript levels of both Hs*ACR3* and Hb*ACR3* compared to the unexposed control (Fig. 6). The highest expression levels were observed at 10 µM As(V), with Hs*ACR3* showing a significantly stronger induction (~180-fold) compared to Hb*ACR3* (~104-fold). At 100 µM As(V), expression levels of both genes decreased substantially.

## Discussion

The arsenic levels detected in *H. bulbiferum* and *H. sinapizans* are consistent with concentrations commonly reported for fruit bodies of other mushrooms, including other *Hebeloma* species, and they are far below the extreme values exceeding 1000 mg kg⁻^1^ dry weight reported for some hyperaccumulating mushrooms (Stijve et al. 1990; Braeuer and Goessler 2019; Borovička et al. 2022). However, the pronounced 20-fold difference in arsenic accumulation between *H. bulbiferum* and *H. sinapizans*, collected at the same site within a few meters of each other, could indicate that these syntopic and phylogenetically related species employ distinct mechanisms of arsenic uptake, transport, or detoxification. Our data indicate that these fungi exhibit distinct capacities for arsenic accumulation and the efflux of As(III) and organoarsenicals (Supplementary Data 1). To elucidate the molecular basis of these traits, we focused on the ACR3 family, as the *S. cerevisiae* transporter ScACR3 represents the sole characterized fungal arsenic efflux system described to date.

The high similarity between HbACR3 and HsACR3 (Fig. 1) suggests conserved functional properties, and their substantial identity 51%, 42%, and 41% with characterized membrane ACR3 transporters from *Pteris cretica var. Albolineata* (Popov et al. 2021), *S. cerevisiae* (Wysocki et al. 1997), and *Corynebacterium glutamicum* (Fu et al. 2009; Villadangos et al. 2012), respectively. This indicates that key mechanisms of arsenite transport are evolutionarily maintained across diverse taxa and further support the evolutionary and functional significance of ACR3 in fungal arsenic metabolism. This notion is reinforced by the conserved cysteinyl and glutamatyl residues identified in HbACR3 and HsACR3, which are likely critical for metalloid translocation (Garbinski et al. 2019) and underscore their role as functional arsenite transporters.

A recent study has shown that *acr3* genes are restricted to certain taxonomic groups, including bacteria, fungi, bryophytes, ferns, and gymnosperms, with the fungal lineage previously underexplored (Biswas and Ganesan 2024). Our findings indicate that ACR3 transporters are widespread within *Agaricomycetes* (Fig. 2A, 2B), a highly diverse class representing a substantial portion of fungal diversity. Modern molecular phylogenetic analyses indicate that *Agaricomycetes*, a class within *Basidiomycota*, is composed of 20 distinct orders and includes more than 21,000 described species, representing one fifth of all known fungi (Hibbett et al. 2014). Despite the availability of genome assemblies for 3278 animal species across 24 phyla (Hotaling et al. 2021) and 631 land plant species (Marks et al. 2021), only a small number of ACR3 orthologs were detected, indicating that their scarcity in these lineages reflects true biological rarity rather than limited genomic sampling. Furthermore, it suggests that higher eukaryotes have evolved alternative As(III) detoxification strategies, such as enzymatic methylation or sequestration via ABC-type transporters (Thomas et al. 2010; Song et al. 2010). Thus, the widespread presence of ACR3 in *Agaricomycetes* suggests it is the primary, ancestral arsenic defense mechanism in this group, contrasting with the different pathways evolved by animals and plants. The observation that ACR3 homologues have been found in so many fungal species, especially from *Agaricomycetes*, could point to the critical, yet overlooked, role of these soil-dwelling fungi as major drivers in the global biogeochemical cycling and mobilization of arsenic.

For functional studies in *S. cerevisiae*, the *arr3*Δ strain was chosen because it carries an inactivated Sc*ARR3* gene, which encodes the plasma membrane efflux transporter required for arsenic resistance and is sequence-related to Hb*ACR3* and Hs*ACR3* (Wysocki et al. 1997; Maciaszczyk-Dziubinska et al. 2011). The functional characterization of Hb*ACR3* and Hs*ACR3* in *S. cerevisiae arr3*Δ mutants (Fig. 3) demonstrates that these fungal transporters can complement arsenic sensitivity, consistent with the role of Sc*ARR3* in arsenite efflux. For example, the *S*. *cerevisiae arr3*Δ mutant was fivefold more sensitive to As(III) and As(V) than the wild-type cells (Wysocki et al. 1997). Similar heterologous expression of the yeast Sc*ARR3* improved the tolerance of *Arabidopsis thaliana* to both As(III) and As(V) (Ali et al. 2012). However, it is important to note that the presence of functional arsenate reductases in yeast and plants can influence the apparent substrate specificity, as As(V) is reduced to As(III) prior to efflux (Dhankher et al. 2006; Mukhopadhyay and Rosen 1998). In contrast, expression of Sc*ARR3* in rice enhanced As(III) efflux and reduced arsenic accumulation in grains, and bacterial ACR3 orthologs appear to be strictly specific for As(III) transport (Duan et al. 2012; Fu et al. 2009; Villadangos et al. 2012). Finally, it should be noted that transport of As(III) has been reported for all characterized ACR3 proteins from plants, bacteria, and yeast, underscoring the conserved function of this transporter family (Fu et al. 2009; Indriolo et al. 2010; Maciaszczyk-Dziubinska et al. 2011; Chen et al. 2017; Popov et al. 2021).

HbACR3 and HsACR3 are localized to the plasma membrane (Fig. 5), like ScARR3 (Wysocki et al. 1997). Nevertheless, it is vital to be aware that mislocalization of heterologous membrane proteins can occur in yeasts. For instance, while PvACR3;1 was localized to the plasma membrane in yeast, it was found at the vacuolar membrane in a plant model (Chen et al. 2017). Similarly, PvACR3 in *P. vittata* was clearly localized to vacuoles (Indriolo et al. 2010). Despite this, several lines of evidence strongly support that HbACR3 and HsACR3 are indeed localizing to the plasma membrane in their native fungal context. First, both HbACR3 and HsACR3 share high protein sequence similarity with ScARR3, a well-characterized plasma membrane efflux transporter (Fig. 1). Second, mycelia of *H. bulbiferum* and *H. sinapizans* actively release iAs into their surrounding medium (Supplementary Data S1), a process consistent with plasma membrane efflux activity rather than vacuolar sequestration. Finally, among all known arsenic efflux protein families, only ACR3 has been described in eukaryotes, while the others are restricted to bacteria and archaea (Shen et al. 2014; Chen et al. 2015, 2016; Shi et al. 2018; Garbinski et al. 2019). Taken together, these findings suggest that HbACR3 and HsACR3 function as plasma membrane arsenic efflux transporters, playing a crucial role in arsenic detoxification in *H. bulbiferum* and *H. sinapizans.*

For the expression analysis of Hb*ACR3* and Hs*ACR3* (Fig. 6), As(V) was chosen as the inducer because it is more prevalent in the natural environment (Liu et al. 2025) and is reduced to As(III) within the fungal cells (Supplementary data S1). A substantial decrease in the expression of both genes at 100 µM As(V) could indicate the activation of alternative detoxification mechanisms, such as arsenic methylation. This process involves the enzymatic conversion of iAs into generally less toxic organic forms through successive methylation steps. These organic arsenic compounds are commonly detected in mushroom fruit bodies, indicating that methylation may play an important role in the natural detoxification of arsenic in mushrooms (Braeuer and Goessler 2019 and references therein), as the organic arsenic compounds were also observed in the *H. bulbiferum* and *H. sinapizans* mycelia in this study. We propose that the limited expression of Hb*ACR3* at lower As(V) levels leads to the intracellular retention of As(III) within *H. bulbiferum*. This retention not only contributes to the increased total arsenic content but likely facilitates downstream methylation by increasing the available substrate pool. Taken together, the differential expression of these transporter genes in mycelia provides a plausible explanation for the variation in arsenic accumulation observed in the fruit bodies.

Given that the accumulation phenotype appears driven by transcriptional regulation rather than protein efficiency, the underlying cause likely lies in the non-coding regions. The hypothesis that regulatory divergence, rather than protein dysfunction, drives the observed phenotypic differences is strongly supported by our heterologous expression data. We observed that Hb*ACR3* and Hs*ACR3* display identical functionality in *S. cerevisiae*, conferring equal levels of arsenic resistance and reducing intracellular accumulation to the same extent. Furthermore, fluorescence microscopy confirmed that both orthologues correctly localize to the plasma membrane. This functional equivalence is consistent with their striking sequence conservation; the two proteins differ by only two amino acids, situated in non-conserved regions (the loop between TMD8–9 and the C-terminal tail) unlikely to affect transport kinetics. Since both proteins are equally competent transporters, the limited Hb*ACR3* induction observed in *H. bulbiferum* mycelia points specifically to variation in upstream *cis*-regulatory elements. Promoter analysis in other arsenite-responsive systems has demonstrated the necessity of specific binding sites for transcriptional activation, such as the Yap8 response element in *Saccharomyces cerevisiae* (Wysocki et al. 2004) or the ArsR operator sites in bacteria (Wu and Rosen 1991). We therefore propose that differences in the promoter architecture of Hb*ACR3* render it less sensitive to arsenite induction compared to the robustly regulated Hs*ACR3*, leading to the observed intracellular retention of arsenic.

## Conclusions

In this study, two fungal ACR3 homologs, Hb*ACR3* and Hs*ACR3*, were identified and functionally characterized. Both genes encode membrane proteins of 399 amino acids that share high sequence identity with known ACR3-type arsenite transporters from other organisms. Phylogenetic and database analyses revealed that ACR3 proteins are predominantly found in mushrooms, particularly within the *Agaricomycetes*, indicating their evolutionary and functional significance in fungal arsenic metabolism. Functional assays in *S. cerevisiae arr3*Δ mutants demonstrated that Hb*ACR3* and Hs*ACR3* restore arsenic resistance and significantly reduce intracellular As(III) accumulation, confirming their role as arsenite efflux transporters. GFP localization experiments further supported their plasma membrane localization, consistent with their efflux function. Moreover, both genes were strongly upregulated upon arsenate exposure, highlighting their involvement in arsenic detoxification. As a conclusion, ACR3 transporters in mushrooms are important for arsenic detoxification, as they mediate arsenite efflux across the plasma membrane and help maintain cellular arsenic homeostasis, protecting the organism from toxic effects.

## Supplementary Information

Below is the link to the electronic supplementary material.

ESM1 (PDF.171 KB)

## Data Availability

No datasets were generated or analyzed during the current study.
