# Supplementary material for: Arsenic efflux mechanisms in ectomycorrhizal mushrooms Hebeloma bulbiferum and Hebeloma sinapizans
Source: Appl Microbiol Biotechnol. 2026 Jan 30;110(1):52. doi: 10.1007/s00253-026-13710-7 (PMC12860867; doi:10.1007/s00253-026-13710-7)
Supplement: Supplementary file 1 — (PDF.171 KB) [file 253_2026_13710_MOESM1_ESM.pdf]

**Supplementary Table 1.** Primers used in this study. The same sets of primers were used for both HsACR3 and HbACR3 amplification		
Primer name	Sequence (5´→3´)	purpose
HsppACR3-p416-InF	TAGAACTAGTGGATCCATGTCTGGAAATGCTTCAGTG	construction of expression vectors
HsppACR3-p416-InR	GCAGCCCGGGGGATCCCTATACAACTTTCTCAGGTG	construction of expression vectors
HsppACR3-GFP-InR	TGCTCACCATGGATCCTACAACTTTCTCAGGTGTCTTCG	GFP-tagged
qHsppACR3-F	GCCAATTGCTGGCCCCTTCA	qPCR
qHsppACR3-R	TGCGATTGGGAGTGAGACGC	qPCR
Hspp-ACR3-GW-F1	CGTATTCCGCGTCTTCGTCCCACTGATCC	genome walking
Hspp-ACR3-GW-F2	CCTCATCGGCCTGCTGTACACCATCATTGT	genome walking
Hspp-ACR3-GW-R1	TTACGGAGTGAGACGCTCTCGAACTTCGCA	genome walking
Hspp-ACR3-GW-R2	GCATTGCGAACGTTGGGTGCAAATTCACCT	genome walking
HspACR3-F3	CAAAGGGCTCAGCATACAGGAC	homology cloning
HspACR3-R4	TCCGAGCTAGATGGTATATCAAC	homology cloning
HsppACR3-DNA-F	AACCCACTCATACCCTCACCAC	amplification of genomic clones
HsppACR3-DNA-R	GGTATTGGGGATAAGAATCTC	amplification of genomic clones
qHspp-TUB2-F	GGAAGTTAGCTGTCAACATGG	amplification of genomic clones
qHspp-TUB2-R	GTGAGTTCAGGAACAGTAACGG	amplification of genomic clones
qHspp-TUB2-F	GGAAGTTAGCTGTCAACATGG	qPCR
qHspp-TUB2-R	GTGAGTTCAGGAACAGTAACGG	qPCR

Supplementary Data S1: Cultivation of *Hebeloma bulbiferum* and *Hebeloma sinapizans* mycelia in liquid PD medium with and without arsenic supplementation for 21 days.

	arsenic supplementation	AsV	AsIII	organoarsenicals
liquid PD medium	-	-	-	-
liquid PD medium	10µM	100%	-	-
liquid PD medium	100µM	100%	-	-
liquid PD medium after cultivation with *H. bulbiferum* mycelium	-	-	-	-
liquid PD medium after cultivation with *H. bulbiferum* mycelium	10µM	84.1%	3.7%	12.3%
liquid PD medium after cultivation with *H. bulbiferum* mycelium	100µM	87.8%	7.3%	4.9%
mycelium *H. bulbiferum* exposed to arsenic	-	-	-	-
mycelium *H. bulbiferum* exposed to arsenic	10µM	8.8%	91.2%	-
mycelium *H. bulbiferum* exposed to arsenic	100µM	50.3%	49.5%	0.2%
liquid PD medium after cultivation with *H. sinapizans* mycelium	-	-	-	-
liquid PD medium after cultivation with *H. sinapizans* mycelium	10µM	8.2%	89.3%	2.5%
liquid PD medium after cultivation with *H. sinapizans* mycelium	100µM	7.1%	92.3%	0.6%
mycelium *H. sinapizans* exposed to arsenic	-	-	-	-
mycelium *H. sinapizans* exposed to arsenic	10µM	36%	64%	-
mycelium *H. sinapizans* exposed to arsenic	100µM	31.8%	67.7%	0.5%
